# Heard it through the grapevine: a phased reference genome for the polyploid Kyoho grape

**DOI:** 10.1093/plphys/kiag378

**Published:** 2026-06-16

**Authors:** William J W Thomas

**Affiliations:** Assisstant Features Editor, Plant Physiology, American Society of Plant Biologists, Rockville, MD, United States; School of Biological Sciences, The University of Western Australia, Perth, Western Australia, Australia

Polyploidy, where more than 2 sets of chromosomes exist in an individual, is a phenomenon that has driven plant evolution. Classical definitions often describe polyploidy as being of 2 kinds: autopolyploidy and allopolyploidy. Autopolyploidy is when a single genome undergoes spontaneous whole-genome duplication, which results in multiple sets of highly similar chromosomes. Allopolyploidy, on the other hand, occurs through interspecific hybridization followed by genome doubling and results in a genome with distinct subgenomes originating from different species. However, many instances of polyploidy do not fit perfectly in either category, and the line between autopolyploidy and allopolyploidy in reality is more blurred than once assumed.

It has been only recently, in the era of modern genomics, that researchers have been able to decipher complex polyploid plant genomes, resulting in an unprecedented understanding of their genomic architecture and evolution (Anderson et al 2026). Genome phasing is the computational technique of separating sets of variants that are inherited together, called haplotypes, based on which parent they originate from. When there is a phased reference genome with haplotype-level resolution, one can answer previously unanswerable questions. For example, which part of the genome came from which ancestral lineage? Are the ancestral genomes distinct or have they mixed over time? Which alleles belong to the same haplotype? The importance of such questions is underscored by the fact that haplotypes are often linked with important agronomic traits, which means phased genomes can facilitate precise haplotype-aware breeding (Bhat et al 2021).

Grapevine is an economically important crop cultivated for wine and table grapes. Most grapevines are the species *Vitis vinifera* because of its large and high-quality fruit, which has suitable chemistry for wine production. However, other *Vitis* species, such as *Vitis labrusca*, have valuable traits such as disease and insect resistance and fruit that has “slip-skins,” where the skins easily separate from the flesh. The “Kyoho” is a Japanese table grape variety created by crossing *V. vinifera* and *V. labrusca*. It was first developed in the 1930s and has since become one of the most cultivated grape varieties, especially in Asia. Unlike most varieties that are diploid, Kyoho is a tetraploid (4 sets of chromosomes), and the architecture of its genome remains largely unexplored.

In a recent study published in *Plant Physiology*, Zhang et al (2026) constructed a near telomere-to-telomere haplotype-resolved reference genome for Kyoho. The genome, sequenced using PacBio HiFi and Oxford Nanopore long-read technologies, was combined with Hi-C scaffolding to assemble the genome into 4 sets of 19 chromosomes. The assembly was highly contiguous with only 11 gaps across all 76 chromosomes. The authors then separated 4 phased copies of each chromosome and assigned them to different haplotypes. It is important to note that the 4 designated haplotypes (hap1–hap4) do not correspond to genome-wide co-segregating haplotypes but are instead used as arbitrary labels to distinguish chromosome-level haplotypes. The 4 haplotypes ranged from 480 to 489 Mb and had BUSCO scores between 97.6% and 98.3%, indicating that each haplotype was highly complete. To characterize the genetic diversity between the 4 haplotypes, they were aligned to the *V. vinifera* reference genome, PNT2T (Shi et al 2023). This identified around 14 million single-nucleotide polymorphisms, 3 million insertions/deletions, and 370,000 structural variants, some of which were haplotype specific. In total, nearly 60,000 genes contained these haplotype-specific variants.

With their phased reference genome, Zhang et al (2026) explored the evolutionary origin of the Kyoho genome. To infer the ancestral contribution of *V. labrusca* and *V. vinifera*, the authors first constructed phylogenomic trees for each chromosome, based on large-scale gene datasets. The phylogenies included related *Vitis* species, as well as both progenitors. Phylogenomic analysis showed that for some chromosomes, the 4 haplotypes originated from the same progenitor, while for others, the haplotypes had mixed ancestry. For example, the 4 haplotypes of chromosome 4 all originated from *V. vinifera*, whereas for chromosome 1, 3 haplotypes originated from *V. vinifera* while the remaining haplotype came from *V. labrusca*. This provided the first line of evidence that the subgenome structure of Kyoho was not straightforward. Next, whole-genome alignments were used to more precisely decipher the genomic landscape. The alignments revealed that the Kyoho genome is a complex mosaic of the ancestral *V. labrusca* and *V. vinifera* genomes (Fig. 1). The mosaic pattern could be observed both at the interchromosome level, where entire chromosomes belonged to different parental haplotypes, and at the intrachromosome level, where segments of DNA displayed chimeric haplotypes originating from both progenitors (Fig. 1c). These results confirm that the Kyoho genome does not neatly separate into 2 distinct ancestral subgenomes; rather, after its formation through interspecific hybridization, it has undergone extensive recombination, resulting in a patchwork-like architecture.

**Figure 1:**
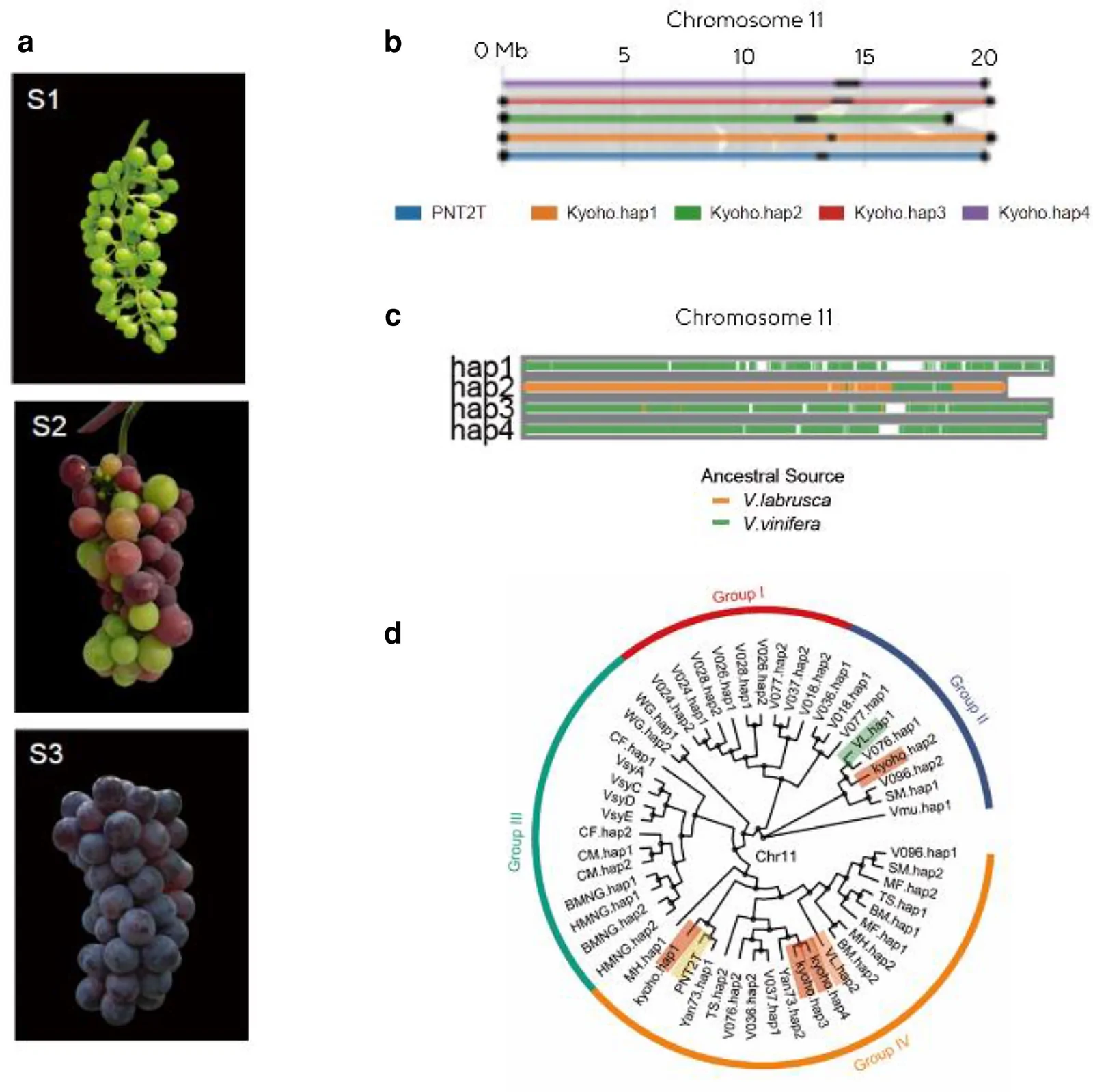
Graphic showing the complex genome architecture in the table grape cultivar Kyoho, with chromosome 11 used as an example. a) The Kyoho grape at different fruit development stages (S1 = young, S2 = beginning of ripening, and S3 = mature). b) The 4 haplotypes of chromosome 11 aligned against the *V. vinifera* reference genome (PNT2T). Black circles at the end of chromosomes represent telomeres, and black boxes in the middle represent centromeres. c) The ancestral source of the 4 chromosome 11 haplotypes showing the genome’s mosaic pattern. Haplotypes 1, 3, and 4 mostly originate from *V. vinifera*, while haplotype 2 originates from *V. labrusca* but also contains recombined segments of the *V. vinifera* ancestral genome. d) The phylogenomic tree for chromosome 11. Haplotypes 1, 3, and 4 cluster closely to PNT2T, while haplotype 2 clusters with *V. labrusca* (VL.hap1). This figure is adapted from Zhang et al. (2026) using components from Figures 1, 2, and S4.

The idea that polyploidy is a spectrum and that there are species that are intermediates between auto- and allopolyploidy has been around for decades (Stebbins 1947). Some of these intermediates were called “segmental allopolyploids” because they contain 2 different ancestral genomes, a feature typical of allopolyploids, but those genomes are similar enough that some chromosomes can still pair and recombine, a feature typical of autopolyploids. Early understanding of segmental allopolyploids was predominantly based on cytological observations of meiotic behavior (Mason and Wendel 2020). It was not until genome phasing that the extent of segmental allopolyploidy could be quantified and its precise haplotype structure identified. There is now a growing number of haplotype-resolved reference genomes that have characterized segmental allopolyploidy in important crops, such as peanut (Bertioli et al 2019), sugarcane (Zhang et al 2025), and sweetpotato (Wu et al 2025). Based on the genomic structure of Kyoho, Zhang et al (2026) classified it as a segmental allopolyploid. By examining the contribution of each ancestral species, the authors found that the Kyoho genome comprised approximately 71% *V. vinifera* and 29% *V. labrusca* ancestry. This highlights that the 2 ancestral genomes have been differentially retained over evolutionary time and are represented unequally within modern-day Kyoho.

Another aspect of the study was to determine how the mosaic ancestry of the Kyoho genome influences gene regulation during fruit development. To do this, Zhang et al (2026) carried out a series of transcriptomic, DNA methylation, and alternative splicing analyses on fruit at 3 different developmental stages (Fig. 1a). Transcriptomic analysis revealed expression bias of alleles originating from either *V. vinifera* or *V. labrusca*, depending on the tissue where the gene was expressed. The authors then performed whole-genome bisulfite sequencing to understand DNA methylation patterns and the epigenetic landscape. A specific type of methylation called CHH, which refers to the methylation of sequences starting with C followed by any 2 bases except G, was found to increase significantly as fruit ripened. The authors suggested that this increase signals the activation of key epigenetic pathways, which help to stabilize the genome during ripening, a process where cells are highly metabolically active and undergo intense biochemical changes. Another interesting finding was that these methylation patterns varied across ancestral genomes, with higher methylation levels detected in genomic segments derived from *V. vinifera* compared to *V. labrusca*.

Finally, the authors assessed the post-transcriptional regulation of genes by investigating alternative splicing. Alternative splicing is the process by which a gene's RNA transcript is combined in different ways to produce more than 1 protein variant, called isoforms, despite having the same gene sequence. Through full-length Iso-seq and short-read RNA-seq, the authors identified a total of 82,750 alternative splicing events in Kyoho. The frequency of alternative splicing events increased as fruit developed, indicating that alternative splicing is an important mechanism driving metabolic change during ripening. Interestingly, homologous gene copies of core ripening regulators exhibited different splicing behaviors, leading to different isoforms. This suggests that ancestral alleles can be processed in different ways to carry out varying specialized functions. Alternative splicing, unlike methylation, was thought to be a key mechanism regulating lineage-specific functional divergence in the Kyoho genome. Collectively, these patterns of gene expression, methylation, and alternative splicing show that functional gene regulation in Kyoho is governed by a highly dynamic, plastic system of developmental context-driven tradeoffs.

The construction of a phased near-complete reference genome for the cultivar Kyoho by Zhang et al (2026) marks a significant step forward in grape genomics. Its genome was revealed to be a segmental allopolyploid with mosaic ancestry from its 2 progenitor species. The reference genome serves as a powerful resource for haplotype-aware modern-day breeding of Kyoho and for understanding the genetic architecture of agronomically important traits in complex polyploid crops.

## Data Availability

No new data were generated or analyzed in support of this article.
